# The safety and efficacy of alfentanil combined with midazolam in fiberoptic bronchoscopy sedation: A randomized, double-blind, controlled trial

**DOI:** 10.3389/fphar.2022.1036840

**Published:** 2022-10-21

**Authors:** Longfei Wang, Qiuyue Wu, Ming Wang, Wanquan Ming, Cheng Sheng, Yonghua Zhang, Yongbin Chen, Yunfei Cao

**Affiliations:** ^1^ School of Medicine, Ningbo University, Ningbo, China; ^2^ Department of Anesthesiology, Beilun District People’s Hospital of Ningbo, Ningbo, China; ^3^ Department of Pulmonary, Beilun District People’s Hospital of Ningbo, Ningbo, China

**Keywords:** Fiberoptic bronchoscopy, Sedation, Alfentanil, Midazolam, Fentanyl, Safety

## Abstract

Sedation is recommended by most guidelines to be offered to all patients undergoing diagnostic flexible bronchoscopy (DFB) without contraindications, and the most commonly reported regimen is midazolam in combination with a short-acting opioid (fentanyl or alfentanil) to provide both sedative and antitussive effects. However, the optimal dose or ideal regimen of the combination therapy with midazolam and opioids has not yet been found. So this randomized, double-blinded clinical trial was designed and registered (ChiCTR2100049052) to assess the safety and efficacy of midazolam combined with different doses of alfentanil in DFB sedation. Our study showed that relative high doses of alfentanil (10–25 μg/kg) combined with a fixed low dose of midazolam can markedly reduce hemodynamic fluctuations, cough reactions, patients’ discomforts, and improve their satisfaction in a dose-dependent manner during DFB, with no significant increase in the desaturation risks.

## Introduction

Though alfentanil is less potent (about five to six times) than fentanyl according to Stanski et al. (Stanski, 1987), it has favorable pharmacological profiles for bronchoscopy (Moman et al., 2022; Loser et al., 2014; Usta et al., 2011), such as rapid onset (1–2 min), short duration (10 min), better antitussive effect, less respiratory depression (therapeutic margin of safety up to 1,080), which appears to offer significant clinical advantages over fentanyl and may therefore be ideal for DFB sedation.

Alfentanil combined with midazolam was the commonest recommended regimen in the guidelines or expert consensus in many countries and regions (Wahidi et al., 2011; Du Rand et al., 2013; Fox et al., 2008; Houghton et al., 2004; Leite et al., 2009), this combination therapy can produce synergistic sedation in patients with FB, but meanwhile may increase potential hypoxia risk (Greig et al., 1995; Pavlin et al., 1996; Peacock et al., 1998; Kodaka et al., 2004; Herrick et al., 1997; Evans et al., 1998). And so far, most of the reported literatures or guidelines (Fox et al., 2008; Ni et al., 2010; Lo et al., 2011) recommended using the combination therapy with relative high dose (more than 0.07 mg/kg) of midazolam and low dose (up to 10 μg/kg) of alfentanil for adult patients, but newly published article (Chen et al., 2022) showed that even combined with propofol, the ED_50_ of alfentanil for suppressing responses to painless bronchoscopy in adult females and males was up to 13.68 ± 4.75 and 17.96 ± 3.45 μg/kg respectively. From this point of view, higher doses of alfentanil are needed to achieve better antitussive and antistress effects for DFB sedation. But till now, there are no reports using the combination regimen of higher-dose alfentanil combined with low-dose midazolam for DFB sedation. Therefore, we performed the current randomized, double-blind study to determine the safety and efficacy of a fixed low dose midazolam combined with different higher doses (10–25 μg/kg) of alfentanil in DFB sedation, which may provide optimized regimen for clinical practice.

## Materials and methods

### Study subjects

This was a randomized, double-blind, controlled study, conducted in a tertiary center after the protocol had been approved by the hospital institutional review board [approval number: 2021-09(YS)]. The clinical trial was registered to chictr.org (ChiCTR2100049052). Written informed consent was obtained from all participants before enrollment.

Through February 2021 to December 2021, patients who had a will to receive intravenous sedation when undergoing diagnostic FB in the endoscopy unit were evaluated for enrollment. A total of 279 adult patients (age range 18–60 years; ASA grade I or II) were recruited and 250 were eligible for this study. Indications for FB included pneumonia (49.6%), bronchiectasis (14.4%), pulmonary shadow (11.6%), hemoptysis (8%), and miscellaneous (16.4%). The exclusion criteria were as follows: 1) Patients with severe sleep apnea syndrome (apnea–hypopnea index > 40) or baseline hypoxia with measured peripheral capillary oxygen saturation (SpO_2_)<90% in room air, 2) patients with a history of alcohol abuse or current use of any psychiatric medication, 3) patients who had neurologic disorders or other conditions contributing to difficulty in assessing a conscious response, 4) patients refusing to give informed consent.

### Procedure and sedation

FB procedure was performed by two experienced bronchoscopists (a chief physician and an associate-chief physician in the department of respiratory at our hospital) using EVIS LUCERA BF-260 series bronchoscope (BF260、BF1T260 and BF-P260F; Olympus; Tokyo, Japan) *via* the nasal route. Diagnostic procedures, including bronchial biopsy, bronchial brushing and bronchoalveolar lavage, were performed according to clinical conditions.

All patients were fasted for 6 h for solids and for 2 h for clear fluids without any pre-medication before the procedure. For the purpose of topical anesthesia, patients all received 5 ml of 2% lidocaine induced by nebulized inhalation for 20 min before operation, and 1 ml of 2% lidocaine solution were applied by nasal swap in each nose after the patients were lying on their dorsal position. All patients received supplemental oxygen *via* nasal cannula (4 L/min).Through an electronic randomization software (Microsoft Excel, Seattle, WA), two hundred and fifty enrolled patients were assigned randomly into five groups containing 50 patients each and the following anesthetic agents were given for sedo-analgesia according to group: Group MF(control group) with fentanyl (1 μg/kg) intravenous infusion 2 min after the administration of midazolam (0.04 mg/kg); Group MA1-MA4 with alfentanil 10,15,20,25 μg/kg intravenous infusion respectively, 2 min after midazolam (0.04 mg/kg) administration. To avoid any memorization bias, a patient could only be induced one time. After induction, nasal cannula (4 L/min) was switched to nasopharynx airway (4 L/min; ID 6.0; Teleflex; Pennsylvania; United States) and then, 5 ml of 2% lidocaine solution was given each time down the bronchoscope with the “spray-as-you-go” technique over the vocal cords and the tracheobronchial tree during fiberoptic bronchoscopy procedure.

Randomization to five different groups was done with a computerized script. Patients were attached with sequential inclusion numbers and randomized into five groups. The computer-generated file was passed un-opened to the nursing staff of the endoscopy unite of our institute. Upon study enrollment the patient was assigned an inclusion number, and the study drug was prepared outside the bronchoscopy suite by an assistant uninvolved in the FB procedure. As the only person who had access to the random list, this unblinded assistant was also responsible for collecting demographic data on patients, getting their written informed consent and preparing the study drugs. The syringe of alfentanil or fentanyl was labelled with the patient’s inclusion number with the same volume (all drugs were dispensed with normal saline to 30 ml) and then passed to the anesthesiologist in charge of sedation. Patients, anesthesiologist in charge of sedation, as well as bronchoscopists were blinded to the sedation regimen. The fentanyl/alfentanil infusion was administered using a same syringe pump at the speed of 20 ml/min (1200 ml/h) 2 min after the administration of midazolam (0.04 mg/kg).

Baseline readings of vital signs (respiratory rate, oxygen saturation, heart rate and noninvasive blood pressure) were obtained before sedation (T0) and continuously monitored until the end of the procedure. Besides T0, clinical data were also recorded at the following time points: 1 min after the administration of drugs (T1), bronchoscope insertion through the glottis to the trachea (T2), bronchoscope removal (at the end of the procedure) (T3), 5 min after the finishment (T4).

Whenever indications of inadequate sedo-analgesia were percepted by bronchoscopist during the procedure, an additional 2 ml of 2% lidocaine was sprayed through the bronchoscope channel to enhance topical anesthesia.1/3 inductive dose of alfentanil will be added 10 min after the first injection if the FB procedure have not finished yet, the amount of drugs for induction before the procedure and the final cumulative doses administrated during the procedure were recorded. Flumazenil would be given at a dose of 5 μg kg^−1^ immediately after the procedure and then, patients were transferred to the recovery room.

On arrival at the recovery room, patients were continuously monitored and received supplemental oxygen (4L/min) through installed nasopharynx airway for 15 min, then transferred to in-patient ward with a 3-h constant monitoring.

### Adverse events and patient assessment

We recorded physiological parameters—heart rate (HR), non-invasive blood pressure (SBP, DBP and MAP) and oxygen saturation (SpO_2_) at baseline (T0) and then four different time points (T1-T4) until the end of the procedure—defined as bronchoscope removal.

We also asked an independent third-part observer to rate his perception of the patient’s severity of cough during the procedure on a 10-point visual analogue scale (VAS) (Leiten et al., 2016) immediately after the FB examination, where 0 represented no cough and 10 represented incessant intolerable cough resulting in procedural interference.

Primary outcome measures were cough response and adverse events. In addition, we recorded episodes of brady- or tachy-cardia (≥ 20% increase or reduction of baseline heart rates, respectively); hyper- or hypo-tension (≥ 20% increase or decrease from baseline mean arterial blood pressure, respectively, hypertension was treated with intravenous urapidil 1 mg per time until BP stabilized); and episodes of hypoxemia, defined as an SpO_2_ <90% for >10s (treated with oxygen supplementation at 6 L·min^−1^ or with verbal and tactile stimulation, chin lifts and temporary manual ventilation).

Secondary outcome measures were postoperative recall, willingness to return, and postoperative discomforts. Postoperative follow-up was performed 6 h after the procedure to avoid any judgement alteration due to residual effect of the drugs. Postoperative recall and willingness to return for a second FB if needed (yes or no) were questioned. Any post-procedure symptoms like lethargy, dizziness, nausea, vomitting were recorded at the same time. This patients’ experience assessment was performed by an independent third-part observer blinded to the type of sedative regimen.

### Outcomes

The primary outcomes were the incidence of hypoxemia and cough severity evaluated by VAS and degree of sedation evaluated by Ramsay sedation scale. The secondary outcomes were the stability of vital signs during the procedure and postoperative recall and willingness to return after the procedure.

### Statistical analysis

Enumeration data is presented as count and proportion and analyzed by chi square test and Fisher exact tests. Measurement data is reported as means ± standard deviation (SD) and treated with the One&Way ANOVA test. Normality of the data distribution was visually assessed by means of histograms. Multiple comparisons among the five groups were computed based on LSD test.

No assumptions were made for missing data and statistical analyses were performed with IBM SPSS Statistics, version 25.0 for Windows, findings were considered statistically significant at *p* less than 0.05.

## Results

### Study population and baseline characteristics

**FIGURE 1:**
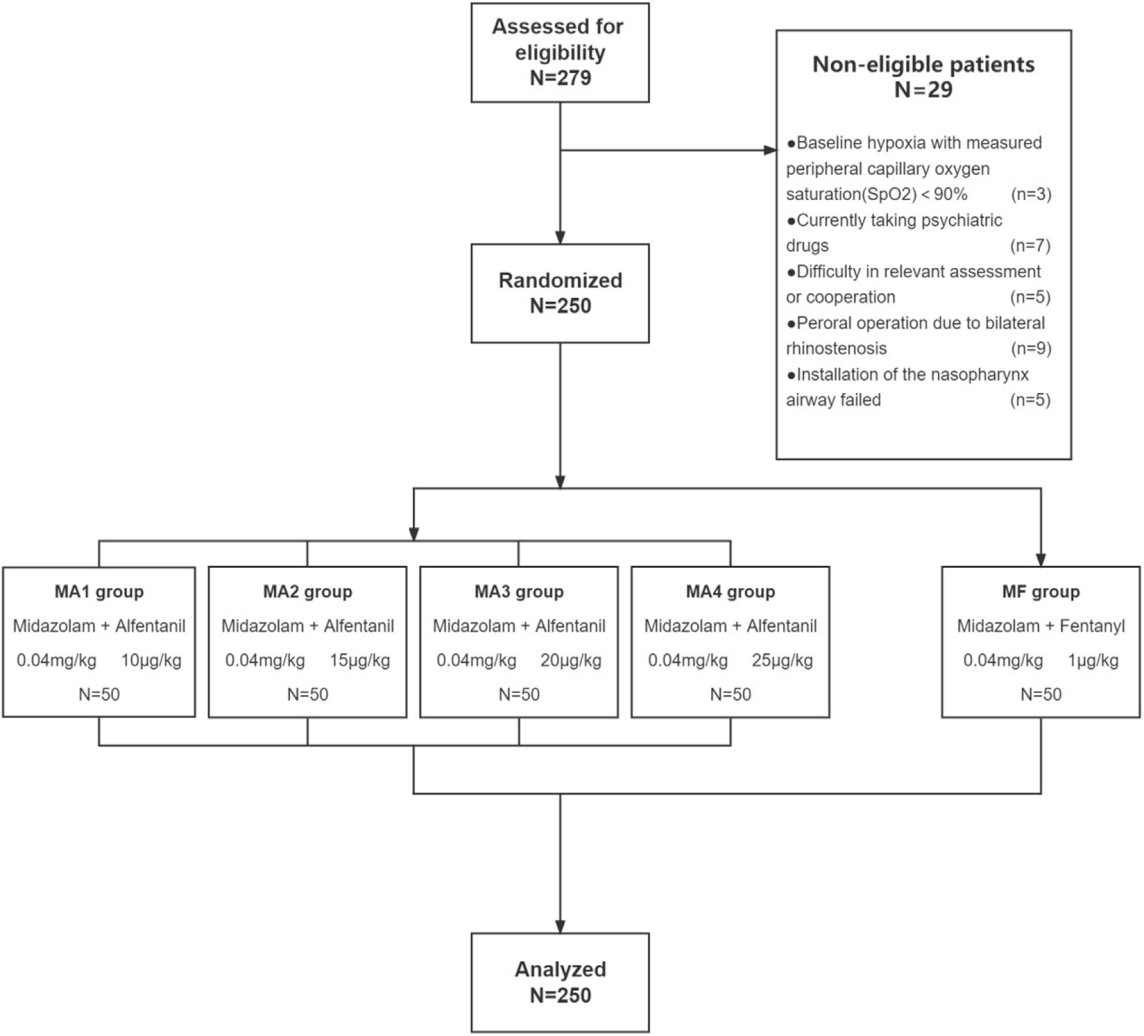
Flow diagram of the study.

Out of screened 279 patients, 250 patients were enrolled in this study and distributed randomly into five groups to receive a fixed-dose of midazolam in combination with four different dosages of alfentanil (group MA1-MA4, *n* = 50) or fentanyl (group MF, *n* = 50) (Figure 1).

In terms of demographic characteristics, there were no significant differences among the five groups with respect to gender, age, weight, height, body mass index, and ASA physical status (*p* > 0.05) (Table 1).

**TABLE 1:** Patient characteristics, Indications and Brochoscopic Procedures (n = 50).

Groups	Group MF	Group MA1	Group MA2	Group MA3	Group MA4	p
**Sex (M/F) (n)**	31/19	28/22	32/18	26/24	27/23	0.705
**Weight (kg)**	65.6 ± 11.8	64.5 ± 12.4	63.8 ± 11.0	63.1 ± 10.3	61.7 ± 10.7	0.499
**Height (cm)**	165.8 ± 8.1	165.5 ± 7.7	165.8 ± 7.9	164.9 ± 7.9	164.7 ± 9.2	0.941
**Age (years)**	43.9 ± 11.6	47.7 ± 10.3	47.3 ± 10.5	45.5 ± 9.6	44.3 ± 12.0	0.287
**BMI (kg/m^2^)**	23.8 ± 3.7	23.4 ± 3.3	23.1 ± 3.1	23.2 ± 2.9	22.6 ± 2.3	0.409
**ASA (I/II) (n)**	41/9	39/11	38/12	43/7	44/6	0.479
**Indications for FB**						
**pneumonia**	28 (56%)	19 (38%)	25 (50%)	28 (56%)	24 (48%)	0.356
**bronchiectasis**	6 (12%)	8 (16%)	8 (16%)	6 (12%)	8 (16%)	0.941
**lung shadow**	3 (6%)	10 (20%)	6 (12%)	5 (10%)	5 (10%)	0.265
**hemoptysis**	5 (10%)	3 (6%)	4 (8%)	2 (4%)	6 (12%)	0.606
**miscellaneous**	8 (16%)	10 (20%)	7 (14%)	9 (18%)	7 (14%)	0.911
**Procedures for FB (n)**						
**BAL**	1	5	6	3	2	0.246
**BAL + BBr**	43	40	35	41	46	0.063
**BAL + BBr + BBi**	6	5	9	6	2	0.280

Data are presented as mean ± SD, or number of patiens(%). BAL, bronchoalveolar lavage; BBr, Bronchial brushing; BBi, Bronchial biopsy.

The primary indication for FB was pneumonia, followed by bronchiectasis, pulmonary shadow and hemoptysis. Diagnostic procedures included bronchoalveolar lavage, bronchial brushing and bronchial biopsy. The indications and procedures were uniformly distributed in five groups (*p* > 0.05) (Table 1).

### Vital signs (MAP and HR) monitoring

For all groups, the variables of vital signs throughout perioperative period at different time points are shown in Table 2. The analysis of MAP and HR showed no statistically significant differences (*p* > 0.05) among five groups at all time points but T2 (*p* < 0.05).

**TABLE 2:** Comparison of the five groups with regard to vital signs(MAP and HR) recorded at different time point during the procedure (n = 50).

Indicators	Groups	Time points				
		T0	T1	T2	T3	T4
**MAP (mmHg)**	**MF (CG)**	98.3 ± 12.4	89.6 ± 10.3	107.5 ± 17.0	96.1 ± 13.2	98.9 ± 14.9
	**MA1 (AF10)**	98.9 ± 14.1	86.6 ± 13.1	100.4 ± 18.0^b^	95.2 ± 13.9	98.9 ± 19.7
	**MA2 (AF15)**	97.8 ± 15.2	84.5 ± 11.5	95.7 ± 12.7^c^	93.6 ± 12.5	91.8 ± 14.6
	**MA3 (AF20)**	95.7 ± 13.0	84.3 ± 13.4	99.1 ± 17.0^c^	95.3 ± 13.5	93.8 ± 15.4
	**MA4 (AF25)**	96.8 ± 14.2	85.7 ± 12.4	96.2 ± 12.7^c^	95.6 ± 11.0	92.6 ± 13.9
		*F* = 0.420	*F* = 1.526	*F* = 4.638	*F* = 0.271	*F* = 2.251
		*p* = 0.794	*p* = 0.195	*p* = 0.001	*p* = 0.896	*p* = 0.064
**HR (beat/min)**	**MF (CG)**	79.5 ± 10.9	74.8 ± 11.7	100.6 ± 13.9	86.4 ± 17.8	82.0 ± 14.4
	**MA1 (AF10)**	81.2 ± 12.1	72.8 ± 12.4	96.4 ± 16.8^a^	88.7 ± 17.8	83.2 ± 12.8
	**MA2 (AF15)**	80.3 ± 15.6	75.7 ± 15.2	95.0 ± 16.7^a^	89.1 ± 18.1	81.7 ± 15.7
	**MA3 (AF20)**	80.8 ± 12.4	73.9 ± 13.2	93.3 ± 14.7^b^	88.4 ± 16.8	82.2 ± 12.0
	**MA4 (AF25)**	80.9 ± 16.2	72.5 ± 14.0	90.8 ± 16.6^c^	84.2 ± 13.9	81.9 ± 15.0
		*F* = 0.117	*F* = 0.513	*F* = 2.670	*F* = 0.717	*F* = 0.086
		*p* = 0.976	*p* = 0.726	*p* = 0.033	*p* = 0.581	*p* = 0.987

Data are presented as mean ± SD. LSD, tests: compared with MF, group, P^a^ > 0.05, P^b^ > 0.05, P^c^ > 0.05. T0: before sedation, T1: 1 min after the administration of drugs, T2: bronchoscope insertion through the glottis to the trachea, T3: bronchoscope removal (at the end of the procedure), T4: 5 min after the finishment.

When compared to values at T2 (bronchoscope insertion through the glottis to the trachea), the data of MAP and HR was significantly higher in MF group (hemodynamic response) than those in other four groups (*p* = 0.001 and *p* = 0.033, respectively). However, no statistical differences were found among the four MA groups (*p* > 0.05).

### Cough response

**FIGURE 2:**
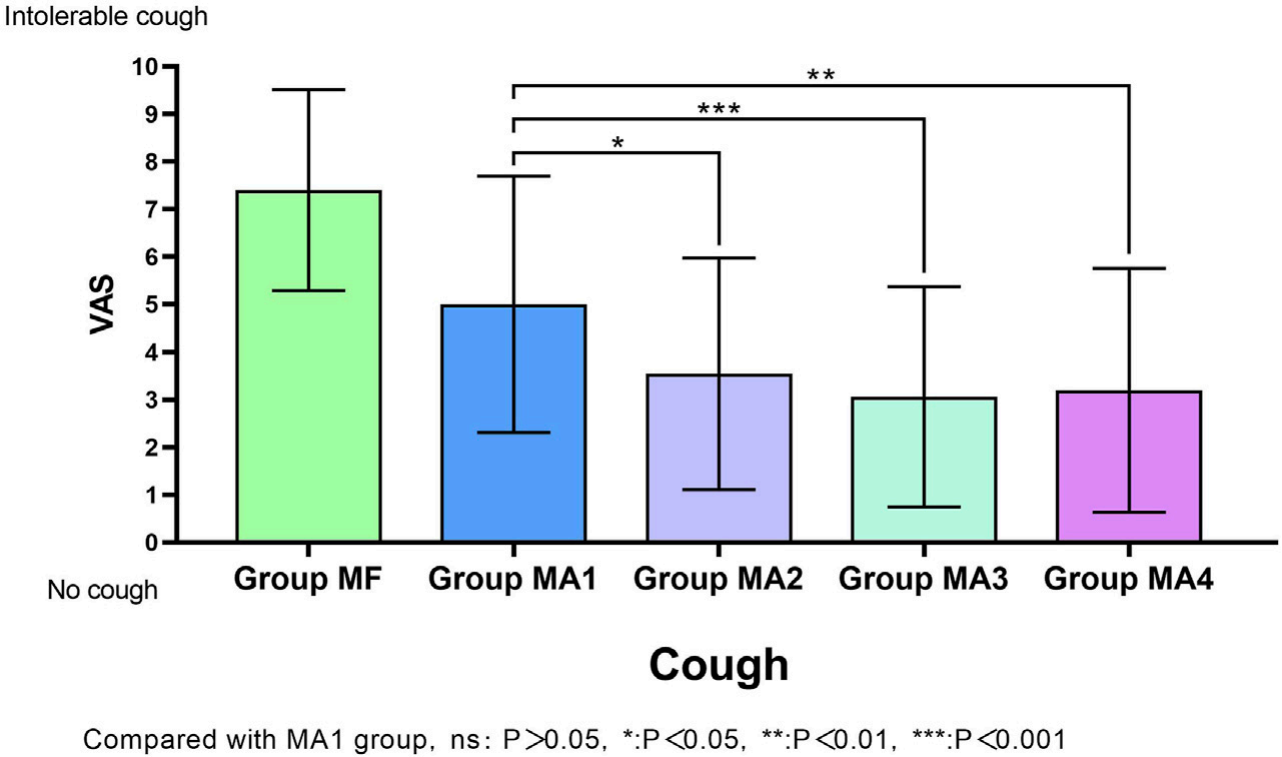
Visual analogue scale (VAS) scores of cough in five groups.

The facts of cough response (including cough incidence and cough severity) for all groups were shown in Table 3 and MA sub-groups in Figure 2. Patients in group MA3 and MA4 had a lower incidence of cough than those in group MF (*p* < 0.05), but no obvious differences were observed in groups MA1 and MA2 when compared with MF (*p* > 0.05).The VAS scores of cough showed significant differences not only between the MF group and the other four groups (*p* = 0.000), but also within the four MA sub-groups, as patients in groups MA2、MA3 and MA4 had a lower VAS scores than that in group MA1 (*p* = 0.0245, *p* = 0.0008 and *p* = 0.0024, respectively).

**TABLE 3:** Comparison of the five groups with regard to cough severity (n = 50).

Groups	Group MF	Group MA1	Group MA2	Group MA3	Group MA4	p
**Incidence of cough**	100%^△^	98%_□_	96%_□_^△^	90%_#_^△^	84%_##_^**^	0.006
	(50/50)	(49/50)	(48/50)	(45/50)	(42/50)	
**Severity of cough** ^‡^	7.4 ± 2.1^***^	5.0 ± 2.7_###_	3.5 ± 2.4_###_^*^	3.1 ± 2.3_###_^***^	3.2 ± 2.6_###_^**^	0.000

LSD, tests: compared with MF, group,*p*_□_>0.05, *p*_#_<0.05, *p*_##_<0.01, *p*_###_<0.001; compared with MA1 group, *p* ^△^>0.05, p*<0.05, p**<0.01, p***<0.001.

^‡^ Assessed by 10-point visual analogue scale (0: no cough; 10: intolerable cough resulting in procedural interference).

### Adverse events

**FIGURE 3:**
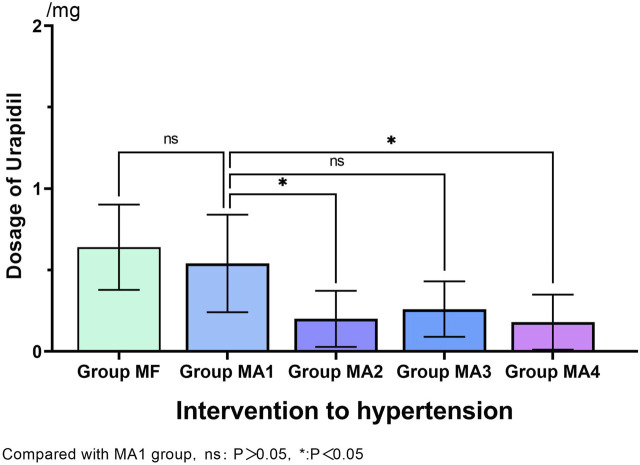
Dosage of Urapidil as intervention to hypertension in five groups.

During the FB operation, the incidence of hypertension (≥20% increase from baseline mean arterial blood pressure) in MF group was 38% (19/50). By comparision, significantly lower incidence of hypertension was observed in MA2、MA3 and MA4 groups (*p* = 0.001、*p* = 0.012 and *p* = 0.000, respectively), but no statistical differences were found between the four MA sub-groups (*p* > 0.05, Table 4). Meanwhile, as an intervention for hypertension, comparison of Urapidil dosages also indicated a similar results. That is, there were significant differences between the MF group and MA2、MA3、MA4 groups, while the dosage of Urapidil in MA2 and MA4 group was significantly lower as compared with that in MA1 group (Figure 3). Although the induction of anesthesia can lead to hypotension in some patients, no vasoactive drugs were used in this study, as the subsequent bronchoscopy could trigger a stress response and reverse the induced hypotension.

**TABLE 4:** Comparison of the five groups with regard to adverse events and clinical interventions (n = 50).

Groups	Group MF	Group MA1	Group MA2	Group MA3	Group MA4	p
**Adverse events**						
**Incidence of hypoxemia**	8%	10%	16%	10%	10%	0.752
	(4/50)	(5/50)	(8/50)	(5/50)	(5/50)	
**Incidence of hypertension**	38%^△^	24%_□_	12%_###_^△^	18%_#_^△^	10%_###_^△^	0.003
	(19/50)	(12/50)	(6/50)	(9/50)	(5/50)	
**Incidence of hypotension**	4%	18%	12%	14%	8%	0.211
	(2/50)	(9/50)	(6/50)	(7/50)	(4/50)	
**Clinical interventions**						
**Dosage of Urapidil (mg)**	0.6 ± 0.9^△^	0.5 ± 1.1_□_	0.2 ± 0.6_##_^*^	0.3 ± 0.6_#_^△^	0.2 ± 0.6_##_^*^	0.006

LSD, tests:compared with MF, group, *p*_□_>0.05, *p*_#_<0.05, *p*_##_<0.01, *p*_###_<0.001; compared with MA1 group, *p* ^△^>0.05, p*<0.05, p**<0.01, p***<0.001.

No statistical differences were found on the incidence of hypoxemia (SpO_2_≤90%). None serious adverse events had ever happened in our study.

### Postoperative follow-up (6 h after the procedure)

There were no differences in postoperative discomforts including lethargy, dizziness, nausea or vomitting (*p* > 0.05, Table 5). But when the patients were questioned about whether postoperative recall exists and the willingness to return for a second FB examination under the same sedo-analgesia, significant differences between MF group (fentanyl group) and four MA sub-groups alfentanil groups, suggest that patients’ satisfaction and comfort in four alfentanil groups were significantly higher than those in the fentanyl group.

**TABLE 5:** Comparison of the five groups with regard to postoperative follow-up (n = 50).

Groups	Group MF	Group MA1	Group MA2	Group MA3	Group MA4	p
**Postoperative discomforts**						
**Nausea**	0%	2%	6%	2%	6%	0.329
	(0/50)	(1/50)	(3/50)	(1/50)	(3/50)	
**Vomiting**	0%	0%	2%	0%	6%	0.070
	(0/50)	(0/50)	(1/50)	(0/50)	(3/50)	
**Dizziness**	16%	16%	18%	8%	14%	0.667
	(8/50)	(8/50)	(9/50)	(4/50)	(7/50)	
**Lethargy**	20%	12%	10%	4%	8%	0.121
	(10/50)	(6/50)	(5/50)	(2/50)	(4/50)	
**Postoperative follow-up**						
**Cases of postoperative recall**	40%	10%***	10%***	8%***	14%***	0.000
	(20/50)	(5/50)	(5/50)	(4/50)	(7/50)	
**Willingness to return**	64%	88%**	96%***	92%***	86%**	0.000
	(32/50)	(44/50)	(48/50)	(46/50)	(43/50)	

LSD, tests: compared with MF, group, p*<0.05, p**<0.01, p***<0.001.

There were more complaining patients in the fentanyl group, and the incidence of postoperative recall was as high as 40% (20/50), which was markedly higher than those in other four MA sub-groups (*p* = 0.000, Table 5). Likewise, with the approval for repetition of the same examination under similar condition, over 85% patients expressed their willingness in MA groups *versus* 64% in MF group (*p* = 0.000, Table 5).

### Indicators related to sedation

**FIGURE 4:**
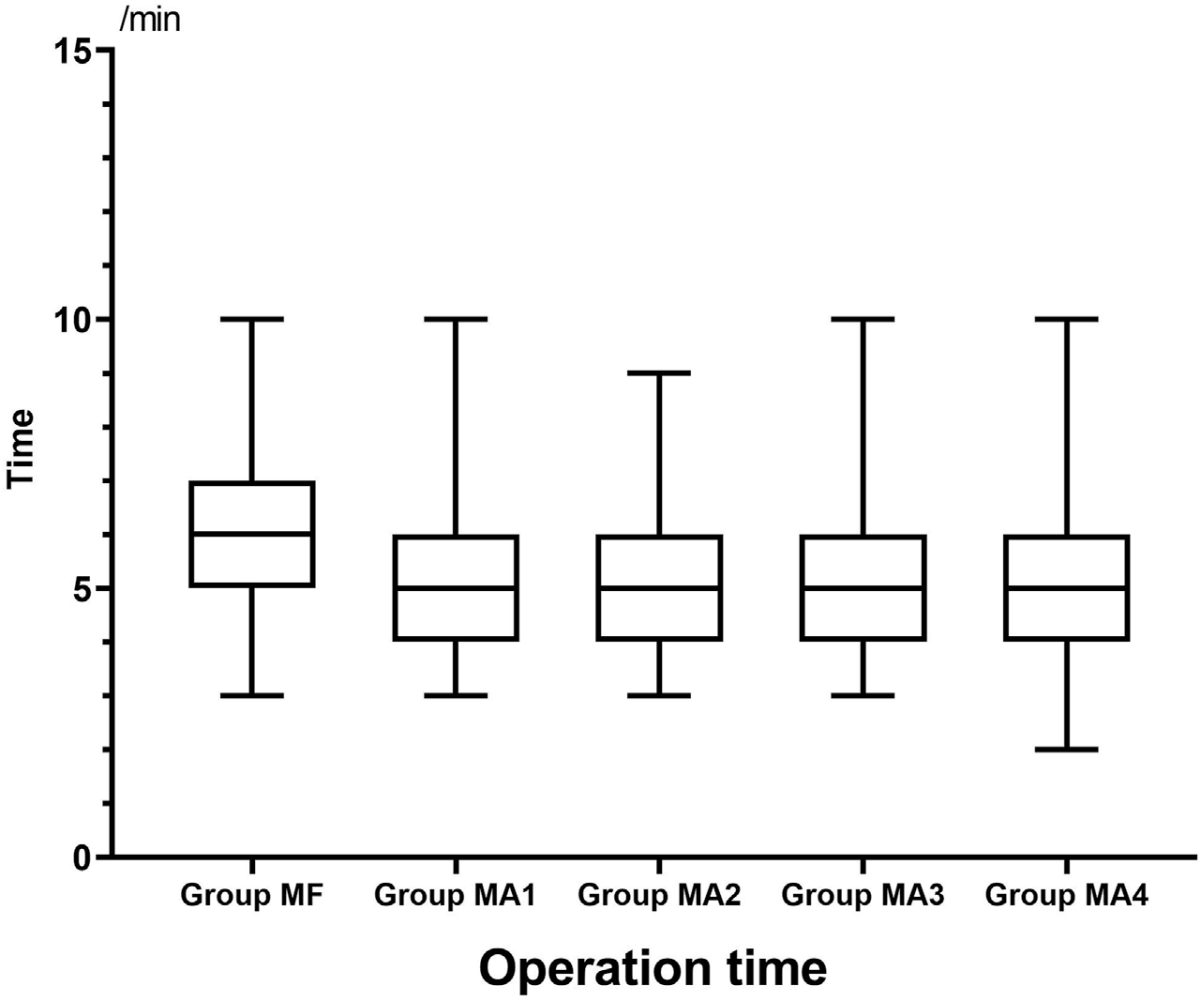
Operation time in five groups.

**FIGURE 5:**
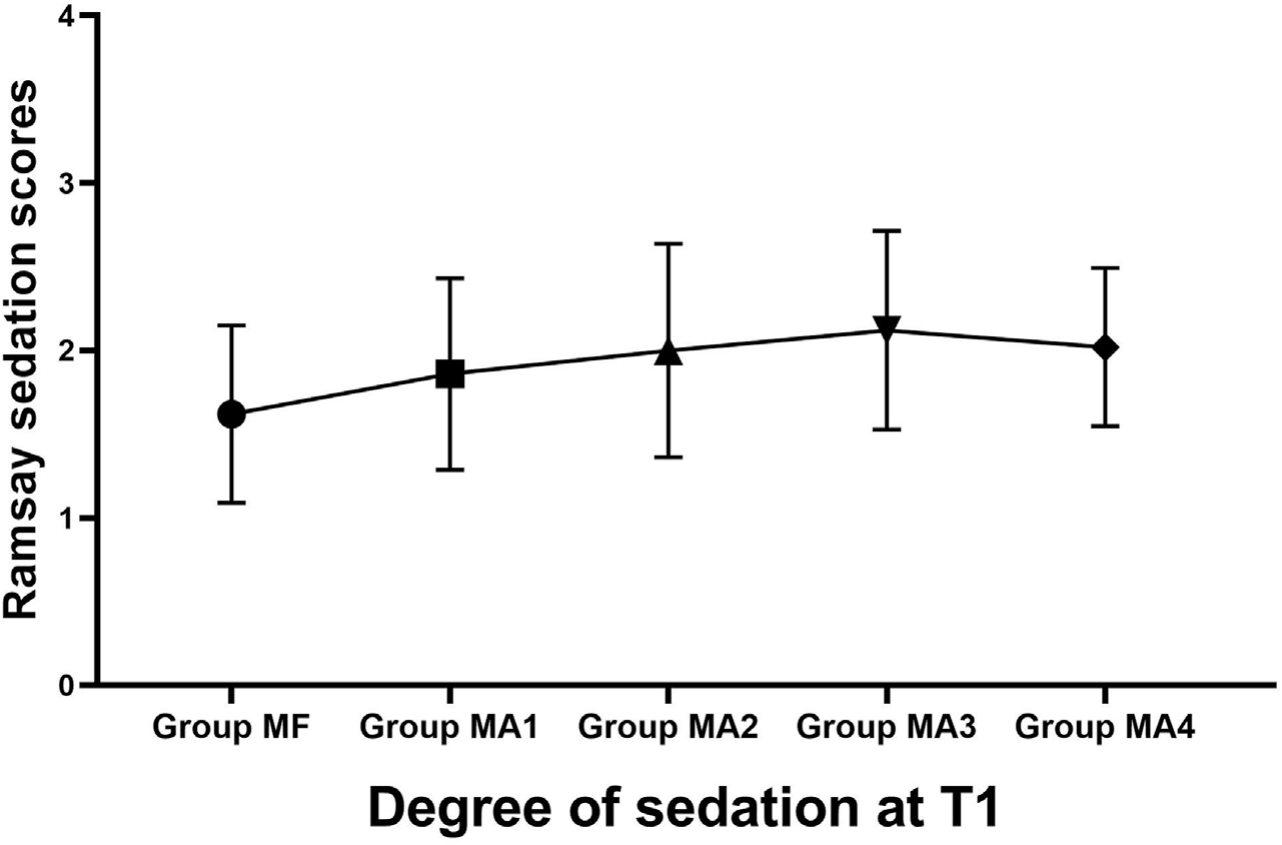
Degree of sedation at T1 in five groups.

**FIGURE 6:**
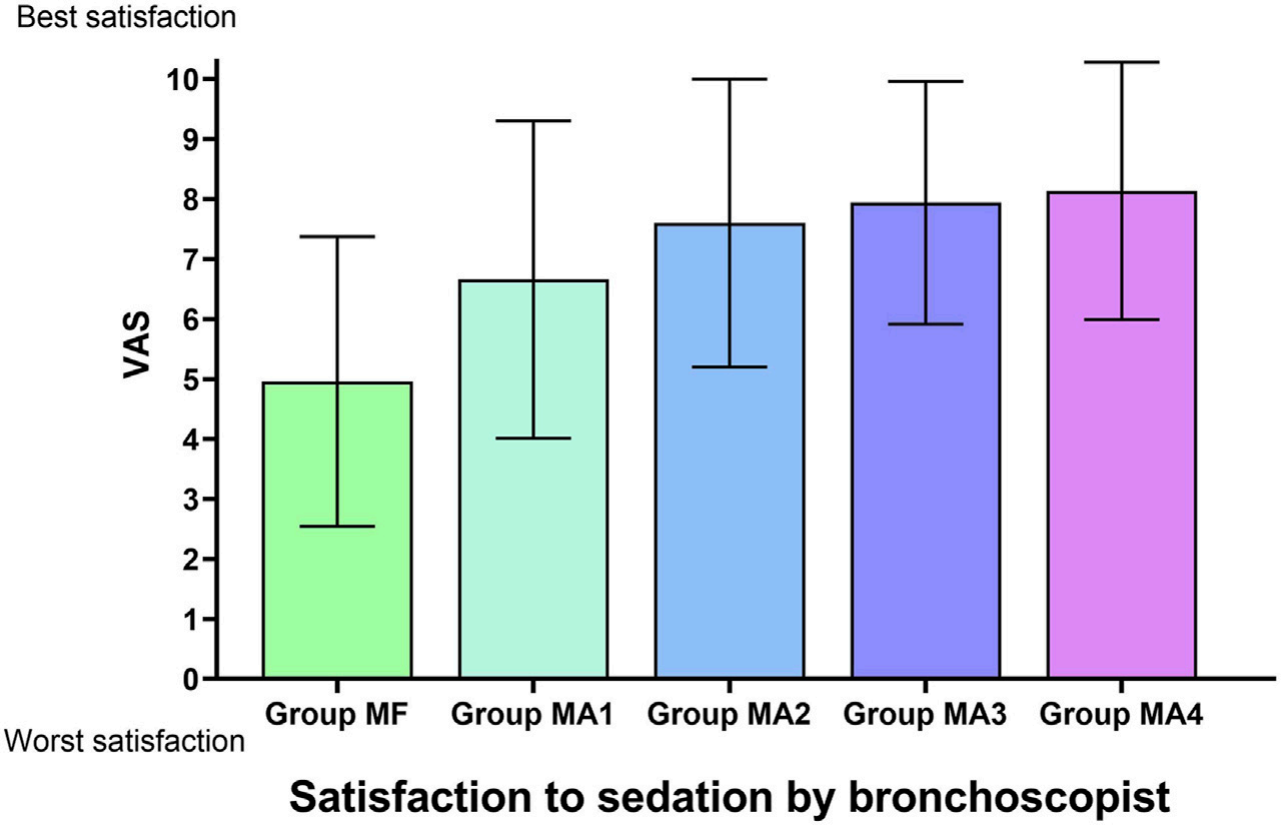
Satisfaction to sedation by bronchoscopist in five groups.

The inter-group analyses showed that MF group had the longest procedure time of diagnostic bronchoscopy (*p* = 0.013, Figure 4 and Table 6), while patients in four MA subgroups experienced higher RSS scores at T1(*p* = 0.000, Figure 5 and Table 6). Bronchoscopists’ satisfaction of sedation anesthesia was also significantly higher in four MA subgroups (*p* = 0.000, Figure 6 and Table 6), and in a dose-related manner.

**TABLE 6:** Comparison of the five groups with regard to indicators related to sedation (n = 50).

Groups	Group MF	Group MA1	Group MA2	Group MA3	Group MA4	p
**Procedure time (min)**	6.0 ± 1.7	5.2 ± 1.5	5.1 ± 1.5	5.0 ± 1.4	5.0 ± 1.6	0.013
**Ramsay sedation scores** ^†^ **at T1**	1.6 ± 0.5*	1.9 ± 0.6_#_	2.0 ± 0.6_##_^△^	2.1 ± 0.6_###_^*^	2.0 ± 0.5_###_^△^	0.000
**Satisfaction to sedation by bronchoscopist** ^‡^	5.0 ± 2.4***	6.7 ± 2.6_###_	7.6 ± 2.4_###_^*^	7.9 ± 2.0_###_^**^	8.1 ± 2.2_###_^**^	0.000

LSD, tests: comlfpared with MF, group, *p*_□_>0.05, *p*_#_<0.05, *p*_##_<0.01, *p*_###_<0.001; compared with MA1 group,*p* ^△^>0.05, p*<0.05, p**<0.01, p***<0.001.

^†^ Ramsay sedatsion scores (RSS):

1.Patient is fully conscious; 2.Patient asleedp but responses to verbal stimulus; 3.Patient asleep but responses to tactile stimulus; 4.Patient asleep, shows no response to verbal or tactile stimulus.

^‡^ Assessed by 10-point visual analogue scale (0: worst satisfaction; 10: best satisfaction).

## Discussion

This study is the first randomized, double blind controlled trial to assess the efficacy and safety of a wider dose range (10–25 μg/kg) of alfentanil combined with a fixed low-dose (0.04 mg/kg) of midazolam in relative healthy patients (ASA class I-II) undergoing diagnostic FB. As compared with midazolam (0.04 mg/kg) -fentanyl (1 μg/kg) combination, the new sedative regime combined with midazolam and alfentanil markedly reduces hemodynamic fluctuations, cough reactions, patients’ discomforts, and improves bronchoscopists’ satisfaction in a dose-dependent manner during DFB. Unexpectedly, the risk of hypoxia did not show a corresponding increase, which suggests that this new sedation regimen may have better prospects for clinical application.

There have been some interesting discoveries in our research. First, up to 10 μg/kg dosage of alfentanil showed similar sedative effect to 1 μg/kg fentanyl in control group, but neither seemed to be sufficient for patients undergoing FB with relative high VAS value of cough (5.0 ± 2.7 vs. 7.4 ± 2.1).Although few cases were found to have postoperative recall after operation, traditional combined sedation with midazolam (0.04 mg/kg) and fentanyl (1 μg/kg) do not appear to be an ideal DBF sedation regime for it is higher VAS scores of cough, instability of circulation and low willingness to return (65%) demonstrated in our study, while midazolam (0.04 mg/kg) combined with different doses of alfentanil (ranged from 10 to 25 μg/kg) showed better sedation and operating conditions in a dose-dependent manner during DFB. To our surprise, there seems to exist ceiling effect on synergistic sedation because no significant differences were found between the two groups with high dose of alfentanil (20 and 25 μg/kg respectively), which indicates that in the clinical practice of adult DBF sedation, the appropriate dose of alfentanil is between 20 and 25 μg/kg when combined with a fixed low-dose (0.04 mg/kg) of midazolam in adult patients.

Second, the intense stress of airway and severe cough irritated by the insertion of bronchoscope into larynx and respiratory tract, was dangerous to patients and may affect the operation of bronchoscope (Chen et al., 2014; Zhang et al., 2019), though patients themselves usually can not feel intraoperatively or recall postoperatively. So in this study, we mainly observed the two indicators of cough response and hemodynamic fluctuation. Although early studies (Webb et al., 1989; Gonzalez et al., 2003; Houghton et al., 2004) have shown that alfentanil has a good antitussive effect on suppressing the cough response during FB, several literatures held different views. According to Yung-Lun Ni et al. (Ni et al., 2010), when patients sedated by the administration of low-dose (5ug/kg) alfentanil combined with relatively large dose of midazolam (5.6 ± 2.6 mg), their cooperation were not significantly better as compared to the control group assessed by the operator’s perception. Moreover, in the research of Greig et al. (Greig et al., 1995), those who received the combination of midazolam and alfentanil had fewer cough counts compared with midazolam alone, no statistical differences were found. This might be related to their low dosage of alfentanil (500 μg), which was insufficient to control patients’ cough response and limb movements caused by FB stimulation. Our study confirmed that higher doses (15–25 μg/kg) of alfentanil was needed to exert better cough suppression, which is consistent with the results of recent literature (Chen et al., 2022) reported that even combined with propofol, the ED_50_ of alfentanil for suppressing responses to painless bronchoscopy in adult females and males was up to 13.68 ± 4.75 and 17.96 ± 3.45 μg/kg respectively. From this point of view, only higher doses of alfentanil can achieve better antitussive and antistress effects for DFB sedation.

Correspondingly, our study showed that higher doses (15–25 μg/kg) of alfentanil combined with midazolam were effective in avoiding circulatory fluctuations (expressed as the incidence of hypertension and the dosage of Urapidil) during FB. The stress of airway irritation during DFB may cause release of catecholamines, hence vasoconstriction, tachycardia, and hypertension (Carmi et al., 2011), and literatures (Fox et al., 2008) showed that alfentanil had the efficacy of obtunding the airway reflexes and attenuating the sympathetic response to FB, at concentration achieved in clinical practice, alfentanil may increase contractile apparatus to cation of calcium (Graham et al., 2004).

Third, respiratory depression and oxygen desaturation are common complications of the sedative medication (Kodaka et al., 2004), our study showed that there existed the risk of hypoxemia (8–16%) whenever midazolam combined with fentanyl or alfentanil. While in some centres, a higher incidence of hypoxemia or desaturate was reported in FB sedation combined with alfentanil and midazolam. According to Uri et al. (Carmi et al., 2011),30.5% and 10.1% patients who received midazolam plus alfentanil needed for oxygen supplementation and airway support respectively. And in studies by Lo et al. (Lo et al., 2011), the ratio of hypoxia display as 35.7% which is fairly similar. However, not all studies agrees with that tendency. Fox et al. (Fox et al., 2008) found only 8.3% of patients had hypoxia occurring which is consistent with our results, though their sedative regime was combined with a higher dose (mean total dose 6.7 mg) of midazolam and a lower dose (625 μg) of alfentanil. Additionally, several recent studies (Dreher et al., 2010; Fruchter et al., 2011) demonstrated that even in patients with pre-existing respiratory failure or severe COPD, midazolam-alfentanil combination during FB sedation was better tolerated by patients without excessive complications on the condition of sufficient oxygen supply and efficient monitoring. Although we used relatively high doses of alfentanil in our study, the dose of midazolam in our combination therapy was significantly lower than that reported in other studies (more than 0.07 mg/kg midazolam usually), which resulted in a small synergistic effect of respiratory depression and acceptable incidence of desaturation. And furthermore, effective oxygen supply *via* nasopharyngeal tube measures we used in this study, was also beneficial to prevent the occurrence of hypoxia to a certain extent.

The present study had several limitations which need to be addressed. First, the sample size was relatively small. However, we were able to detect statistically significant differences among all groups towards variety of observation index, confirming that the sample size was sufficient to detect clinically important differences in this practice. Second, the potency ratio for fentanyl:alfentanil of 10:1 was chosen because of the convenient to calculate in a clinical setting. However, this ratio is not equipotent. According to Stanski et al. (Stanski, 1987), alfentanil is five to six times less potent than fentanyl. Finally, the safety observed with combined midazolam and alfentanil administration is confined to relatively healthy adult patients undergoing diagnostic bronchoscopic procedures. These results cannot be extended to relatively difficult ultrasound needle aspiration bronchoscopy or interventional procedures, which can be lengthy and may require deeper sedation (Lee and Mathur, 2010). Meanwhile, the findings of the present study are only valid for relatively healthy patients, therefore, generalization of the findings to other patients, (i.e. elderly patient or with pre-existing respiratory failure), is supposed to be limited.

In conclusion, we have discovered a new regimen combined with low-dose midazolam (0.04 μg/kg) and relative high dose (15–25 μg/kg) of alfentanil used for FB in adults Chinese population, which produces a preferable sedation effect and with no significant decrease in safety. Given the relatively small sample size, the results of this study should be adopted after validation by studies with larger samples.

## Data Availability

The datasets presented in this study can be found in online repositories. The names of the repository/repositories and accession number(s) can be found below: The original datasets for this study can be found in the FigShare (https://figshare.com/s/859eedb7f25d71ff0ff4).
